# Application of ATR-FTIR Incorporated with Multivariate Data Analysis for Discrimination and Quantification of Urea as an Adulterant in UHT Milk

**DOI:** 10.3390/foods12152855

**Published:** 2023-07-27

**Authors:** Emeline Tan, Norliza Binti Julmohammad, Wee Yin Koh, Muhamad Shirwan Abdullah Sani, Babak Rasti

**Affiliations:** 1 Faculty of Food Science and Nutrition, Universiti Malaysia Sabah, Kota Kinabalu 88400, Sabah, Malaysia; emeline_tan_mn21@iluv.ums.edu.my (E.T.); weeyin@ums.edu.my (W.Y.K.); rasti_babak@yahoo.com (B.R.); 2 International Institute for Halal Research and Training, Level 3, KICT Building, International Islamic University Malaysia, Jalan Gombak, Kuala Lumpur 53100, Malaysia; shirwansany@iium.edu.my

**Keywords:** milk fraud, adulterants, urea, Fourier transform–infrared spectroscopy, discriminant, regression analysis

## Abstract

Urea is naturally present in milk, yet urea is added intentionally to increase milk’s nitrogen content and shelf life. In this study, a total of 50 Ultra heat treatment (UHT) milk samples were spiked with known urea concentrations (0–5 *w*/*v*%). Attenuated total reflectance–Fourier transform infrared (ATR-FTIR) spectroscopy with principal component analysis (PCA), discriminant analysis (DA), and multiple linear regression (MLR) were used for the discrimination and quantification of urea. The PCA was built using 387 variables with higher FL > 0.75 from the first PCA with cumulative variability (90.036%). Subsequently, the DA model was built using the same variables from PCA and demonstrated the good distinction between unadulterated and adulterated milk, with a correct classification rate of 98% for cross-validation. The MLR model used 48 variables with *p*-value < 0.05 from the DA model and gave R^2^ values greater than 0.90, with RMSE and MSE below 1 for cross-validation and prediction. The DA and MLR models were then validated externally using a test dataset, which shows 100% correct classification, and the *t*-test result (*p* > 0.05) indicated that the MLR could determine the percentage of urea in UHT milk within the permission limit (70 mg/mL). In short, the wavenumbers 1626.63, 1601.98, and 1585.5534 cm^−1^ are suitable as fingerprint regions for detecting urea in UHT milk.

## 1. Introduction

Milk has received considerable attention from global dairy organisations due to its protein content, which contributes to the functional properties of the milk. Antanasova and Ivanova [1] have reported that milk proteins benefit human health, such as anti-microbial, immunomodulatory, anti-thrombotic, antihypertensive activities, and antioxidative properties. Therefore, there is rising demand—in Malaysia in 2020, the domestic milk consumption was 69.4 million litres per year. Moreover, the subsistence level (SSL) for fresh milk is targeted to increase from 62.4% in 2020 to 100% by 2030 under the National Agro Food Policy 2021–2030 [2]. Ultra heat treatment (UHT) liquid milk has become popular due to its long consumption period. UHT is a heat treatment process used to sterilise raw milk and extend its shelf life for up to 9 months at room temperature. During the UHT process, the milk is heated for a split second or two at temperatures ranging from 130 to 140 degrees Celsius (°C) [3]. Subsequently, the milk is homogenised by an aseptic homogeniser to distribute the fat globules evenly and prevent cream formation. Thus, UHT milk is simpler to analyse than raw milk, which has complex constituents.

Therefore, this scenario has enabled certain markets to manipulate milk’s protein composition by adding hazardous substances, including urea. There are two methods by which milk can be contaminated with urea: deliberate inclusion of urea and the addition of unspecified synthetic milk, which imitates pure milk and contains other adulterants besides urea. This class of adulteration is prevalent because non-protein nitrogen cannot be differentiated by the Kjedahl and Dumas methods, which are commonly used to determine the total protein content in dairy products. Urea is a naturally occurring constituent of milk derived from the grass or feed consumed by dairy cattle. The concentration of urea in milk is influenced by various factors, including season, parity group, lactation stage, and milk collection time, as Godden et al. [4] reported. Thus, a standard permissible limit of 10–16 mg/dL for urea in milk has been established, as noted by Abdallah et al. [5], for consumer safety. Whereas, The Food Safety and Standards Authority of India (FSSAI) has set a maximum limit of 70 mg/100 mL [6,7]. Above this limit, urea is presumed to have been added externally, as excessive urea in milk can harm the public, especially pregnant women, children, and individuals with compromised immune systems. Health hazards such as acidity, indigestion, ulcers, and cancer are associated with these conditions. Moreover, Francis et al. [8] assert that urea has detrimental effects on the cardiac system, particularly the liver, as it necessitates increased renal workload to eliminate urea from the body.

Hence, many researchers developed an official non-protein detection method—chromatography with good sensitivity [9,10,11]. However, in terms of sample preparation, this method is complicated. The estimated time for processing each sample, including column cleaning, is 16 min. Moreover, they require the utilisation of expensive chemical substances and specialised expertise. Dutta et al. [12] have recently introduced a method for determining urea using nano-silver particles and a spectrophotometer. This method has demonstrated the ability to measure urea concentration within acceptable limits. However, it requires an extraction step, characterisation of silver nanoparticles, and preparation of the stock sample.

Alternatively, spectroscopic techniques are well recognised as solvent-free, quick, and simple tools for performing chemical analyses on a wide range of matrices without causing harm to the samples and are environmentally friendly [8,13]. This fingerprint method, particularly Fourier transform infrared (FTIR), is possibly the best method when combined with multivariate analysis tools for fast and reliable detection and quantification of various adulterants in milk and has been studied for the past decades [14,15,16,17,18,19]. In addition, the attenuated total reflection (FTIR) technique has been gaining interest for urea detection in UHT milk mainly due to its high analytical capacity, minimal sample processing, and ability to analyse many samples simultaneously [20]. Moreover, utilising an ATR cell rather than a KBr pellet simplifies sample preparation, as the penetration depth in the sample of IR radiation is independent of sample thickness [21].

According to a survey of academic literature on Google Scholar, Web of Science, and Scopus, researchers, including Mabood et al. [22], Amsaraj et al. [23], and Conceição et al. [24], have conducted studies on urea using vibrational spectroscopy techniques and regression analysis methods such as partial least square regression (PLSR) and principal component regression (PCR). Iqbal et al. [25] employed a chemical-based test and Fourier transform infrared spectroscopy (FTIR). The statistical tools utilised in the study included the Chi-Square test and ANOVA statistical analysis. Jha et al. [26] employed SIMCA for classification and multiple linear regression (MLR) for quantification. The model detection efficiency was 86%, which included relatively smaller samples. Furthermore, this study has not used the complete spectra range in the exploratory and regression framework.

Other than the statistical part, the study of adulterants in UHT milk is scarce, as Grassi et al. [21] argue that a study on commercial samples from local grocery stores could be inappropriate as they have already passed to technological operation. However, Souza et al. [27] discovered several adulterants in Brazilian UHT milk. Furthermore, Jeyaletchumi et al. [28] explained the safety of chocolate-flavoured UHT milk to school children who encountered problems due to microbiological contamination along the supply chain in Sabah, Malaysia. Additionally, the UHT method encourages inherent alterations; it does not affect milk’s nutritional value or adulterations. Therefore, even after processing, milk adulteration can be identified [29]. The study of physiochemical changes in UHT milk by Grewal et al. [30] also mentioned that the aggregation of milk proteins progressed slowly during storage at room temperature and did not much affect the spectral data of protein FTIR. 

Therefore, this study aims to explore the potential of discriminant analysis (DA) and MLR coupled with ATR-FTIR as an alternative and a logical method for the non-dangerous, simple, and fast quantitative determination of different urea concentrations in UHT milk. Moreover, this study used another smaller spectral range chosen based on the previous study [26], which was 1675–1560 cm^−1^ and 1585–1454 cm^−1^. Previous studies have found that these wavenumbers were useful for quantifying urea in milk, as these regions showed apparent differences in absorption values of milk and adulterated milk. For comparison, our study used the full spectra (4000–500 cm^−1^) and let PCA and DA models choose the important wavenumbers for the MLR model. The performance between different spectral ranges was evaluated based on the figures of merits of the DA and MLR models. Initially, the process of data pre-processing was executed, which involved the assessment of missing data and the elimination of outliers. Subsequently, all datasets underwent variable transformation before analysis by multivariate models. The suitability of the dataset for multivariate data analysis was evaluated using the Kaiser–Meyer–Olkin (KMO) measure. Then, the DA classification and MLR quantification models were formulated and assessed using established performance indicators, including sensitivity, specificity, and efficiency rates for the discriminant model and R^2^, RMSE, and MSE for the regression model.

## 2. Materials and Method

### 2.1. Experimental Design

**Figure 1:**
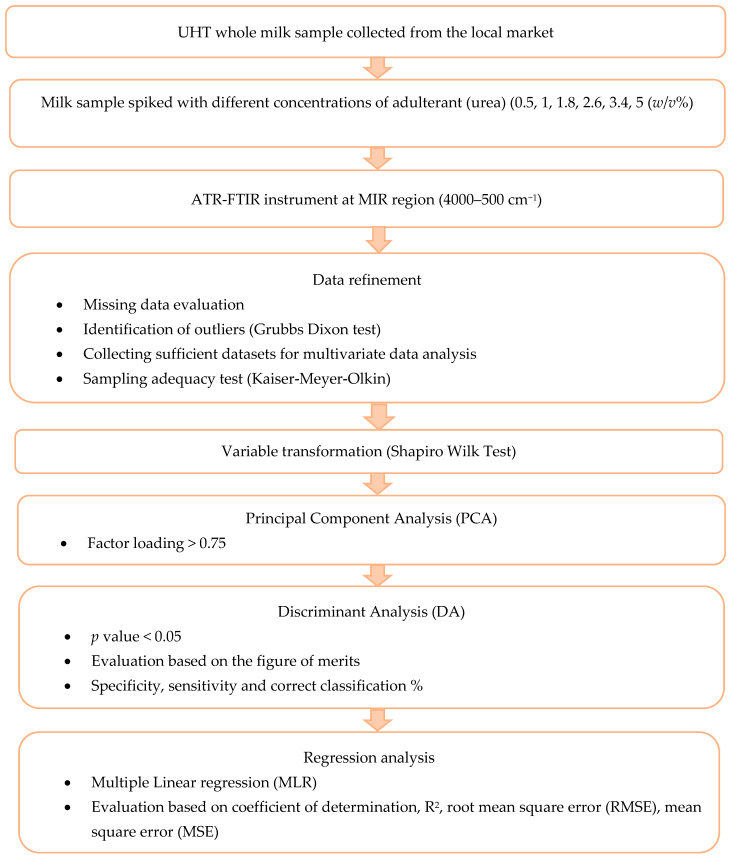
Experimental design of identification and quantification of urea in UHT milk.

This section provides a comprehensive overview of how the experiment’s objective progresses. Figure 1 displays the classification and prediction plan. Further elaboration on the operational details is provided in the subsequent section.

### 2.2. Materials and Sample Preparation

A total of 50 samples of the local brand UHT whole milk were purchased at the local market and spiked with the known concentration of urea. The urea used was of analytical grade by Sigma Aldrich, Merck, Burlington, MA, USA, The composition of the nutritional information for UHT milk, including carbohydrates, lactose, protein milk solids (non-fat), milk fats, sodium, iodine, and vitamins (A, D3, B2, and B12), is displayed on the packaging. The milk sample was spiked with urea at the following concentrations: 0, 0.5, 1, 1.8, 2.6, 3.4, 4.2, and 5 (*w*/*v*%), whereby the samples with 0% urea concentration were assumed as control or pure milk samples. These concentrations are within the allowable limit set up by FSSAI (70 mg/mL) for urea in liquid milk [31]. All samples were vortexed for 5 s to mix the adulterant well with the milk. The milk samples were stored in cold storage (±8 °C) before analysis on the same day the sample was prepared.

### 2.3. FTIR Spectral Acquisition

Before the analysis, a performance test was performed on the mid-infrared Fourier transform infrared–attenuated total reflection (FTIR-ATR) of Bruker Alpha II, Billerica, MA, USA. The background spectrum was scanned at the start of the measurements with an empty diamond ATR cell in the range of 4000 to 500 cm^−1^ at 16 scans and a nominal resolution of 4 cm^−1^. All samples’ spectra were analysed directly without pre-treatment using the same instrumental conditions where approximately 30 µL were placed on the ATR cell. The background spectrum was deducted from the sample spectrum to obtain the actual sample’s range. After each sample analysis, the ATR cell was cleaned with distilled water and ethanol; a background scan was also performed to ensure a correct representation of the sample’s spectrum. The FTIR spectral data in the mid-infrared (MIR) region (4000–500 cm^−1^) of all the pure and adulterated milk were gathered, resulting in 200 spectra (7 concentration × 25 spectra replication), including 25 pure milk spectra. The average spectrum of 16 scans was used, and the spectra acquisition mode was transmittance. The spectra were then exported from the OPUS software (Bruker, Alpha II) to CSV file format and subjected to subsequent chemometric analysis using XLSTAT 2022 (Addinsoft, Paris, France).

### 2.4. Dataset Refinement

The spectral data were exported to Microsoft Excel for data analysis using the XLSTAT (version 2023.1.6) statistical software developed by Addinsoft, Paris, France. The dataset was divided into two groups: the training and testing datasets using the Paretto principle (80:20 ratio) [32,33]. At the same time, the cross-validation dataset consists of 40% of the training dataset, which brought 160 samples × 426 wavenumbers in the training dataset and 64 samples × 426 wavenumbers for cross-validation using the leave-one-out cross-validation (LOOCV) method. Approximately 200 spectral data points, including the control sample, underwent further pre-processing data analyses, such as assessment of transformations, removal of missing data, identification of outliers, variable transformation, and the KMO. Table 1 summarises the number of samples prepared and the 80 to 20 ratio of training and testing datasets [34].

### 2.5. Evaluation of Outlier

The dataset was subjected to an outlier assessment. An outlier is characterised as a variable that displays a significantly deviant value from the overall values. Identifying and confirming outliers within the group were conducted using a standardised dataset. The Grubbs test by XLSTAT 2022 (Addinsoft, Paris, France) detected any missing data and identified the outliers. The outlier’s outcomes were determined using a Monte Carlo simulation approach, which yielded a *p*-value < 0.0001, indicating that the null hypothesis should be rejected in favour of the alternative hypothesis, suggesting the presence of an outlier within the replication of the sample. In addition, the Z-test score was also provided, in which the test computes the number of standard deviations by which the data varies from the mean. For more information on the Grubbs, the test can be found in a paper review by [35].

### 2.6. Variable Transformation

To verify the normal distribution of the dataset before conducting PCA, a Shapiro–Wilk (SWT) test by XLSTAT 2022 software (Addinsoft, Paris, France) was performed with a significance level of α = 0.05. The dataset underwent a standardisation (n − 1) method. This transformation of spectral data variables aims to maintain a higher similarity between the transformed and original datasets [36].

### 2.7. Principal Component Analysis (PCA)

The present study utilised the training dataset to conduct a PCA analysis based on Pearson correlation by XLSTAT 2022 software (Addinsoft, Paris, France). The aim was to investigate the dataset pattern, evaluate the spectra data’s role in classifying pure and adulterated milk, elucidate the correlation among the spectra data, as well as effectively reduce the dataset (*p* < 0.05) into smaller sets of new independent variables, which is referred to as principal components (PCs), as per the following Equation (1):

(1) $$X={TP}^{T}+E$$

*X* represents the (n × m) of measurement or predictors. n is the number of UHT milk samples, whereas m is the wavenumbers. Superscript *T* represent a transpose operation, and (n × l) score matrix is a projection of the *X* (the *X* score, component or factor matrix). *P* represents (m × l) orthogonal loading matrix, respectively, and *E* is the residual matrix. Its entries are assumed to be independent and identically distributed standard random variables, and l is the number of significant or retained PCs [37,38]

Two PCAs were conducted in this study. The initial PCA involved the complete spectral range of 4000–500 cm^−1^, and statistical measures such as cumulative variability (CV), eigenvalue (EV), KMO test, and Bartlett’s test of sphericity (FL) were assessed at a significance level (α) of 0.05. The second PCA utilised spectral data that had factor loading (FL) > 0.75 [39]. The first PCA was compared to subsequent CV, eigenvalue, KMO, and FL evaluations. The study evaluated the FL and spectral correlation and investigated the allocation of spectra to the urea concentration. The significant variable for urea concentration was proposed to be the selected spectral data from the second principal component analysis [40].

Visualising the PCA output involves the creation of a scatterplot, wherein the position of each sample was plotted around zero based on its corresponding value of principal components (PCs) [41]. PCA is frequently employed as a screening technique to help visualise differences between a pure sample and an adulterated sample [42]. Moreover, using PCA enables the acquisition of uncorrelated variables, thus eliminating multicollinearity, which often happens for spectral data as a number of variables is bigger than the number of variables. Reducing multicollinearity effects is crucial to achieving accurate quantification using the MLR model [43].

### 2.8. Discriminant Analysis (DA)

Discriminant analysis is a common method for descriptive and predictive data analysis. This study employed DA as a supervised technique to delineate and elucidate the distinctions between pure milk and a set of adulterated milk samples. The process of DA involves determining the original variables’ linear combination that maximises the distance between the group as per mathematical principles. Discriminant analysis may encounter variables with zero variance or multicollinearity among variables. XLSTAT by Addinsoft, Paris, France, avoids these issues by automatically excluding the variable from computations. In this study, the discriminant model was executed at set α of 0.05 and conducted as the covariance matrices are identical using Equation (2) and significant variables obtained from the PCA:

(2) $$f\left(C_{a}\right)=K_{a}+\sum_{a=1}^{n}\left(W_{a}{\cdot T}_{a}\right)$$

where *a* represents the number of milk clusters (*C*); *K* represents the constant for each cluster; *T* denotes the classification of the training dataset into the cluster; *n* represents the number of wavenumbers, and *W* represents the weight coefficient.

In this section, the spectra of 200 samples, including pure and impure milk, were split into two sets 80% for the training dataset and 20% for the testing dataset (Table 1). Significant variables identified by PCA and two smaller spectra, 1675–1560 cm^−1^ and 1585–1454 cm^−1^, were used to compare the classification performance such as specificity, sensitivity, and efficiency, which were calculated following Equations (3) and (4):

(3) $$\mathrm{Sensitivity}\;(\%)=\frac{TP}{TP+FN}\cdot 100$$

(4) $$\mathrm{Specificity}\;(\%)=\frac{TN}{TN+FP}\cdot 100$$

Sensitivity (Equation (3)) refers to its accuracy in identifying the proportion of samples belonging to the modelled class. In contrast, specificity relates to the ratio of samples from other categories that the model correctly rejects. The evaluation of the classification performance was based on four categories: true positive (*TP*) identifications, which adulterated samples correctly classified as such; true negative (*TN*) identifications, which refer to unadulterated samples correctly classified as unadulterated; false negative (*FN*) identifications, which refer to adulterated samples incorrectly classified pure; and false positive (*FP*) identifications, which refer to pure samples incorrectly classified as adulterated. Different assessment parameters, efficiency, are defined as the geometric mean of specificity and sensitivity, which ranges from 0 to 1 [44].

(5) $$\mathrm{Efficiency}\;(\%)=\sqrt{\frac{TP\cdot TN}{\left(TP+FN\right)\cdot (TN+FP)}}\cdot 100$$

As DA is a supervised method, a new column labelled “adulterants” was added to the dataset, in which pure milk was assigned as “pure milk”, and different concentrations of urea in milk were set as “milk + urea”. The correct classification was carried out on the cross-validation and externally validated by testing the dataset. The significant variables were identified based on a unidimensional test of equality based on Wilk’s univariate lambda is between 0–1, a lower value of *p* < 0.05, meaning there are common intra-class variations and therefore high inter-class variations (XLSTAT 2022, Addinsoft, Paris, France). The regression model did not include remaining variables higher than *p* = 0.05.

### 2.9. Regression Analysis

The urea levels were measured using multivariate regression, starting from the lowest concentration to the highest. This model enabled a mathematical function to relate the response variables to the explanatory variables used to predict the outcomes. MLR is a simple multivariate regression technique that aims to represent the relationship between two or more explanatory variables (an NK matrix’s columns belong to a single vector) and a response. Variable (y is an N 1 vector) is determined by fitting an ordinary least squares regression (y = Xb; b is calculated by solving b = (X X) − 1 X y) [45]. The spectral data used for the MLR model were based on the PCA result with factor loading FL > 0.75 followed by a variable with a *p*-value lower than 0.05 in the DA section, which brought 160 samples × 48 wavenumbers. The aim was to minimise the data matrix’s dimension, eliminate the variables’ multicollinearity, and keep the model from overfitting. Model performance was evaluated through training, cross-validation, and prediction on the testing dataset, with 64 of the training datasets used for cross-validation; otherwise, 35 of the test datapoints that did not belong to the training datasets were used for the prediction test.

Like the DA section, two different wavenumbers were utilised based on fingerprint regions selected by previous studies by Jha et al. [26] and Conceicao et al. [24]. The model is generally scored based on the R^2^ closest to 1, and the lowest RMSEC and MSE indicate that the selected model has the lowest probability (1%) of incorrect predictive values. A supplementary column titled “urea percentage” was incorporated into the training dataset within this particular section. The formula for calculating the prediction of urea is below (Equation (6)), where *C* is the regression coefficient value, *N*_1_ is the first independent variable, and *C_n_ + N_n_* is the regression coefficient of the last independent variable.

(Urea %) = Intercept + (*C*_1_·*N*_1_) + (*C*_2_·*N*_2_) + … (*C_n_·N_n_*) (6)

## 3. Results and Discussion

### 3.1. Identification of Functional Groups of Urea in UHT Milk Based on FTIR Spectra

**Figure 2:**
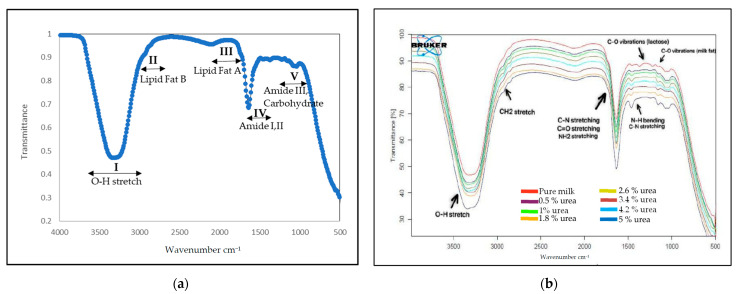
(**a**) FTIR spectrum for blank UHT milk at wavenumber 4000−500 cm^−1^. (**b**) FTIR spectrum for unadulterated UHT milk and different concentrations of urea (0%, 0.5%, 1%, 1.8%, 2.6%, 3.4%, 4.2% and 5%) spiked in UHT milk at wavenumber 4000−500 cm^−1^. The presence of 5% (*w*/*v*) urea in milk can be seen at regions 1700−1500 cm^−1^ and 1472−1239 cm^−1^.

Figure 2 shows the average FTIR spectra of pure and adulterated UHT milk (0.5%–5 *w*/*v*%) (b) within the 4000–500 cm^−1^ region. The spectral data did not undergo any spectral pre-processing of the MIR spectra to avoid the loss of any peak that might contribute to the identification of urea in the milk sample. The UHT milk region can be categorised into five distinct regions, as illustrated in Figure 2a. These regions include region I, 3700–3000 cm^−1^, and are associated with hydrogen bonding related to water. Region II, ranging from 3000 to 2800 cm^−1^, is linked to lipids (fat B). Region III, which spans from 1800–1700 cm^−1^, is also associated with lipids (fat A). Region IV, ranging from 1700 to 1500 cm^−1^, is linked to amide I and II; region V, spanning from 1500 to 900 cm^−1^, is associated with amide III and carbohydrates [46].

The broad peak observed in region I is attributed to the O-H bond in water, which can be related to the liquid state of the UHT milk sample. Region II is attributed to fat B, featuring peaks at 2926 cm^−1^ and 2857 cm^−1^, corresponding to the asymmetric and symmetric stretching modes of the CH_2_ groups present in the acyl chains of milk lipids, respectively, triglycerides and phospholipids. Region III is attributed to fat A, wherein the C=O stretching vibrations of ester linkages of triacylglycerols are linked to the protein–lipid association through carbonyl groups, with a wavenumber range of 1755–1752 cm^−1^ [47]. This study’s primary focus region is Region IV, which pertains to the protein group found in milk, specifically casein and whey protein. The spectral regions of Amide I (1700–1600 cm^−1^) and Amide II (1600–1500 cm^−1^) indicate C=O stretching vibrations of the peptide bonds and C-N stretching vibrations in conjunction with N-H bending, respectively. These vibrations provide valuable insights into the secondary structure of proteins [48]. Region V is associated with amide III and carbohydrates, particularly lactose, found in milk. Figure 2b shows a spectrum of a sample adulterated with 5% (*w*/*v*) of urea, which shows an apparent variance in absorption frequencies observed between pure and adulterated milk at 1670–1564 cm^−1^. This disparity can be attributed to the protein region close to N-H bonding, as evidenced by the intensity of the signal. Thus, this spectral region indicates the presence of CO, CN, and NH_2_ vibrations [25,49]. Additionally, the presence of urea more visible at higher concentrations in milk was associated with changes in the protein vibrations at 1700–1500 cm^−1^ (amide I and II) and 1472–1239 cm^−1^ (Amide III) [50]. The results of the current study were comparable with those in the literature.

However, in lower urea concentrations, the intensity of the spectral changes was barely noticeable and overlapped significantly. In addition, it has been reported that urea is naturally present in milk, making it difficult to distinguish it unequivocally from a sole spectrum dependency. Plus, the amide is a crucial protein group marker, and several types of amides with different protein samples may result in overlapping or weakening the transmittance values [25,51]. Therefore, it is necessary to use mathematical spectral transformations to extract meaningful information for qualitative and quantitative analyses [52]. The classification of the pure and adulterated UHT milk was further analysed by PCA and DA, and the quantification through the MLR model.

### 3.2. Determination of Significant Wavenumbers for Adulterated UHT Milk via Principal Component Analysis (PCA)

As described in the methods, PCA was used as an unsupervised method to classify the milk sample based on the correlation between the variable. Before the analysis, all observations (outliers) were removed from the dataset. The KMO verified the sampling adequacy as a statistic, indicating the proportion of variance underlying factors might cause in the variable. In the food authentication field, KMO values (close to 1) indicate that factor analysis may be helpful for the data. Otherwise, with a value of less than 0.5, the result of the factor analysis may not be beneficial [53]. Meanwhile, Bartlett’s sphericity was used to test the hypothesis that the correlation matrix is an identity matrix to determine whether the variables are unrelated and, therefore, are unsuitable for structure detection. Smaller than 0.05 significance level values indicate the usefulness of the factor analysis in the dataset.

**Figure 3:**
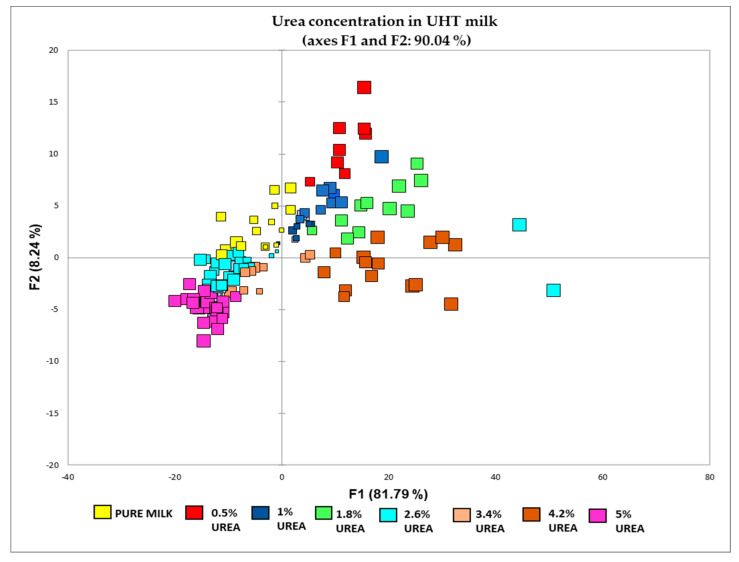
PCA for classification of pure milk and different concentration of urea (0.5%, 1%, 1.8%, 2.6%, 3.4%, 4.2%, and 5%) in UHT milk.

In this study, two PCA were carried out. The first PCA using the entire spectra (4000–500 cm^−1^) showed KMO 0.956 and PC1 and PC2 with eigenvalues (EV) > 1 [54], which explained the dataset’s 85.29% cumulative variability (CV). The variable from the first PCA with strong factor loading (FL > 0.75) was used to build the second PCA, as these variables significantly contribute to the calculation of the PC axis. Therefore, this particular tool can be utilised to select variables [55]. Compared to the first PCA, this PCA showed greater PC1 and PC2 with CV (90.036) and KMO (0.960). This result confirmed that 386 variables adequately explained the urea concentration variation in the UHT milk sample after data transformation. Even though no cut-off value for factor loading exists, variables with VF coefficients of |0.500|, FL |0.749|, and FL |0.499| were considered to have moderate and weak factor loadings, respectively [56]. The score plot of the second PCA (Figure 3) shows a spectrum showing eight distinct groups, including the pure and urea-adulterated UHT milk samples. They are arranged and grouped in the specific regions of the PCA score plot. All the urea-adulterated milk samples appear in the right regions of the PCA score plot compared to pure milk samples. The position of pure milk and 5% urea concentration in milk is opposite, making these two groups negatively correlated, indicating that a positive change of pure milk spectra will not affect 5% urea and vice versa. Based on the exploratory data analysis, the statistical plots within the range indicated that all could be used in regression analysis [57]. Even though all the outliers have been treated through the outlier inspection, close observation via exploratory data analysis reveals an irregular pattern. This outlier is only visible in a 2-dimensional space, as shown in Figure 3; two observations were far from the centroids and could not be classified correctly. However, the outlier did not significantly affect the exploratory data analysis; hence, it can be left as it is. As stated by Data et al. [58], none of the outlier removal techniques can improve the performance of a classification model. Extreme values are probably due to biological variation rather than experimental mistakes.

However, it can be noticed that some of the group overlaps with other concentrations. Visualising the PCA using the entire spectra in this study is complex due to the high number of variables. There are also several guidelines to ensure PCA is appropriate for the data by providing multiple variables are measured continuously. In addition, there must be a linear relationship between the variables, which can be checked using scatter plots. PCA cannot always solve multicollinearity-related problems with parameter estimation by multicollinearity [59]. Therefore, discriminant analysis (DA) was employed to reduce the insignificant variable for further analysis by MLR.

### 3.3. Classification of Adulterated UHT Milk via Discriminant Analysis (DA)

DA is a commonly utilised supervised method for food analysis, including authentication, characterisation, and adulteration detection, with vibrational spectroscopy as an instrument used. This approach has been documented in various studies, such as those conducted by [60,61,62,63]. Even though it has been reported that DA may encounter difficulties identifying a lower dimensional space when the dimensionality is significantly higher than the number of samples in the data matrix, commonly called the small sample problem. This issue can be resolved by utilising an intermediate subspace, such as PCA, to transform the within-class matrix into a matrix with total rank, thereby enabling its inversion [64]. In addition, the DA was used to help reduce the multicollinearity within the variable and discriminate the sample based on its group (pure or adulterated). The DA in this study was performed on the training and testing dataset, as mentioned in Table 1. There were three different approaches for modelling the DA: 386 variables (4000–500 cm^−1^), 15 variables (1675–1560 cm^−1^), and 17 variables (1585–1454 cm^−1^). The aims of this part were to observe which approaches give higher specificity and sensitivity for discrimination between the sample group [24,26,64]. The DA score plot obtained from PCA > 0.75, indicates a very good separation between pure milk and the mixture of urea + milk. Based on Fisher’s distance, the *p*-values for both groups (pure and adulterated milk) were less than 0.05, indicating that a significant difference correctly developed between both groups. These differences were confirmed by the Wilks Lambda test, which showed a *p*-value < 0.0001, indicating that the two sample groups differed. The DA selected the significant variable in this analysis based on a *p*-value < 0.05. Cross-validation and prediction using a testing dataset were performed to verify the discrimination model. 

Based on Table 2, the DA model achieved a 100% classification rate in the training dataset using PCA variables for pure and adulterated milk. Similarly, the cross-validation demonstrated a precise classification of pure milk with 100% accuracy. However, 98.73% was obtained for adulterated milk, suggesting one adulterated milk was incorrectly classified as pure. Meanwhile, for the spectral range of 1675–1560 cm^−1^ and 1585–1454 cm^−1^, the DA model accurately classified adulterated milk but not pure milk. Similar outcomes were noted for the cross-validation results. For the testing dataset, the DA model can correctly predict the class of the sample for the variable chosen by the PCA; in contrast, DA showed poor performance in predicting the sample using two smaller spectral regions. Compared with a previous study, Jha et al. [26] utilised SIMCA to classify various urea concentrations in cow milk using a similar wavenumber range. They only achieved 80–98%, and none reached 100% classification efficiency (%), similar to the value achieved for wavenumber 1675–1560 cm^−1^ and 1585–1454 cm^−1^ utilised in this study. Meanwhile, Rani Amsaraj et al. [23] used PLS-DA to classify urea and other adulterants, where the result was 0.999 for both calibration and prediction.

Above all, using the variable selected from PCA achieved higher sensitivity and specificity for the DA model. Furthermore, none of the adulterated samples was predicted as pure milk and vice versa for training and testing datasets except for cross-validation. Even so, a 100% correct classification was recorded for testing, thus proving the reliability of the DA model for discriminant and prediction of unknown samples. Conversely, lower correct classification was achieved for training, cross-validation, and testing for the wavenumber region at 1675–1560 cm^−1^ and 1585–1454 cm^−1^, indicating that this spectral range cannot optimise the DA model performance. The DA model also reduces the variables by selecting a new significant one based on *p* < 0.05 from the total 386 variables proposed by the second PCA to 48 variables. The model allows the 48 variables to be 95% confident to be selected as the significant variables in this study. The predictive performance of the dataset (Table 2) demonstrated the DA model’s capability to classify pure and adulterated milk samples correctly.

### 3.4. Quantification of Urea Adulteration in UHT Milk Regression Analysis

As stated in the methodology section, urea content in milk was quantified using MLR. This study utilised 48 significant variables selected through DA to build the MLR model. Subsequently, a comparison was made with the smaller spectral regions, 1675–1560 cm^−1^ (15 variables) and 1585–1453 cm^−1^ (17 variables). All spectral windows exhibited R^2^ values exceeding 0.9 for both calibration and cross-validation. The spectral windows of 1675–1560 cm^−1^ showed the highest R^2^ values of 0.991 and 0.933 for calibration and cross-validation, respectively. Meanwhile, 1675–1560 cm^−1^ reached 0.99 and 0.981, respectively, followed by the variable selected by PCA and DA model, which achieved 0.996 and 0.940, respectively. Furthermore, it was observed that the RMSEC and MSE values were lower in the spectral range of 1675–1560 cm^−1^, with respective values of 0.170 and 0.029. This trend was also observed during cross-validation, with values of 0.168 and 0.028 for RMSE and MSE, respectively, indicating improved prediction stability. Similarly, other spectral regions also showed a lower relative error, which was below 1. Based on Table 3, each of the spectral ranges identified the most significant wavenumber to detect urea in UHT milk. The significant wavenumbers chosen were 1626.63, 1601.98, and 1585.5534 cm^−1^, which agrees with the urea absorption peak observed in FTIR. This considerable variable is related to the presence of urease protein [56] and CO stretching in urea. At the same time, 1585.5534 cm^−1^ is associated with C-N stretching vibrations and NH_2_ bending vibrations related to amide III [26].

Based on Table 4, the determined value for each concentration was within the actual value. The specified value also fell between the determined value’s 95% lower and upper bounds. Among the spectral ranges, significant variables from the DA model showed an excellent prediction compared to others based on the determined adulteration and *t*-test value. However, this chosen wavenumber cannot predict urea concentration in milk as low as 0.5% as the null hypothesis was rejected and the alternative hypothesis accepted, indicating the differences between the actual and prediction mean are different from 0. Nevertheless, 1 *w*/*v*% of urea in milk still can be accepted; thus, this model can be used to quantify urea in UHT milk despite the complexity of the UHT milk composition, as 1% is still within the permissible limit of urea. Negatively predicted values for 0% concentration have been observed for the spectral ranges of 1675–1560 cm^−1^ and 1585–1485 cm^−1^. This pattern was also seen by Idris et al. [34], where the prediction of lard adulteration using PLSR also had a negative value for 0% lard, similar to Santos et al. [65] for the quantification of urea in milk. It can be reasonably and legitimately replaced with a 0 value if the interval’s lower point is negative. This will maintain the confidence level. However, the estimated outputs can be checked against the actual values by looking at the *t*-test value. Both spectral ranges at 0% concentration have *p*-value < 0.0001, indicating that the difference between the mean is different. Thus, the MLR model using these spectral regions incorrectly predicts the unadulterated milk sample. The reason might be an incomplete or inadequate dataset, as the regression analysis relies on having a representative and sufficient dataset for accurate predictions.

**Figure 4:**
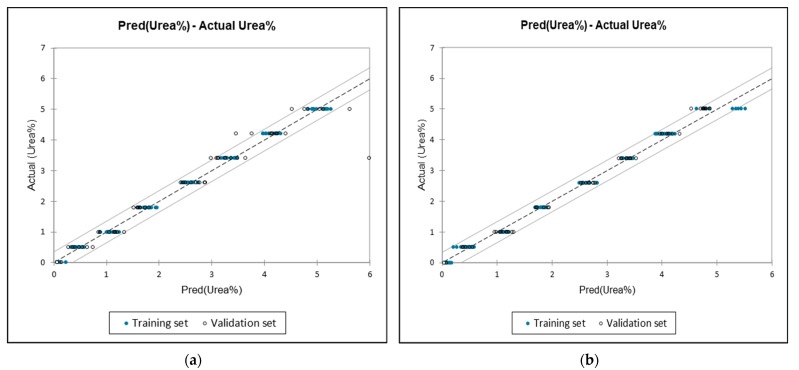

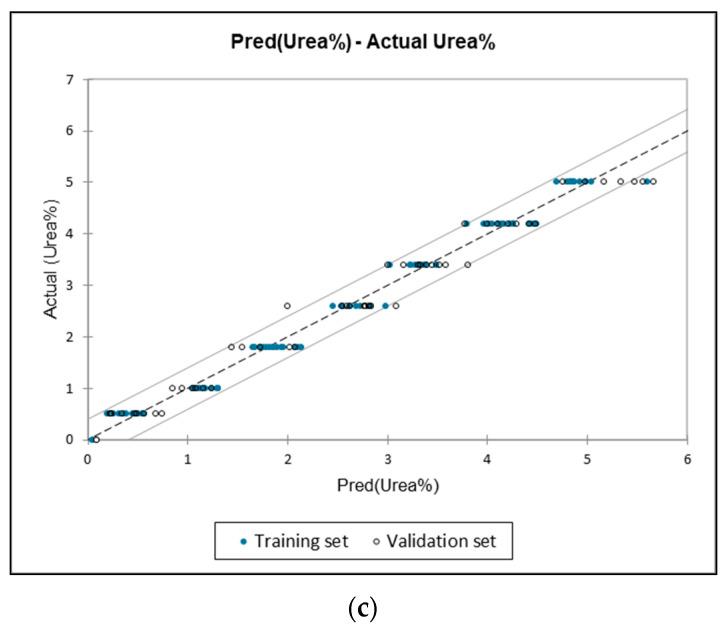
(**a**) Actual vs. predicted levels of added urea content in milk (4000–500 cm^−1^). (**b**) Actual vs. predicted levels of added urea content in milk (1675–1560 cm^−1^). (**c**) Actual vs. predicted levels of added urea content in milk (1585–1454 cm^−1^).

The MLR scatterplot depicting all spectral ranges (Figure 4) indicates a good concordance between the reference values and the predicted FTIR values for urea bias, as evidenced by the R^2^, RMSEC, and MSE metrics. Our study’s results compare favourably to those reported by Basak et al. [52], who employed PLSR with a pre-processing technique known as multiplicative scatter correction (MSC) to quantify urea in milk, which achieved R^2^ = 0.99, with an RMSE value of 3.35, which is higher than our study, which achieved below 1. They also found the presence of urea in six brands of commercial samples, as urea is naturally present in milk, but none of the samples contained higher than the permissible limit. As with Jha et al. [26], the optimal prediction of urea quantity was achieved using PLSR in the spectral range of 1649–1621 and 1611–1580 cm^−1^, resulting in R^2^ values of 0.906 and 0.879 for calibration and validation, respectively. Alternatively, MLR resulted in R^2^ values of 0.931 and 0.867 for calibration and validation, respectively. Both models exhibited a higher RMSE when compared to the results of our study. At the same time, Conceicao et al. [24] employed MLR to detect the presence of urea in raw milk. The authors achieved R^2^ values of 0.80 and 0.72 for the training and validation sets, respectively. A lower R^2^ was achieved by Khishor and Thakur [66] by the utilisation of PLSR with values R^2^ of 0.279 and 0.188 for calibration and validation, respectively, with lower relative error values. In contrast, the study conducted by Amsaraj et al. [23] utilised PLS2-based regression and showed that the calibration and prediction achieved values above 0.90, respectively, with a relative error lower than 1 for quantification of urea, starch, and sucrose.

Overall, the MLR model with minimum error and the highest R^2^ was the best. In this context, three different wavenumbers gave good prediction values. Even so, the *t*-test value for full spectra showed very similarly predicted with the actual concentration value; thus, the MLR model was suitable to be used based on the reduction of variables using PCA, and DA utilised the entire FTIR spectra and let the PCA and DA model choose the spectral range compared to the assumed spectral range based on the spectra peak of 1675–1560 cm^−1^ and 1585–1454 cm^−1^. Although MLR is reportedly not ideal for use with FTIR spectral data, it can lead to high multicollinearity, increased overfitting, and reduced model robustness [45]. Yet, several studies [24,46,62,67] employed MLR with other regression models, and MLR yields competitive outcomes and sometimes outperforms PCR and PLSR models. All of the mentioned studies utilised a limited spectral range determined by the notable absorption peak observed and subsequently implemented the MLR model. Instead, our study used PCA and DA to help lessen the variable and help reduce the multicollinearity between the variables [43]. Nonetheless, Grassi et al. [21] emphasise that the power analysis could be performed to develop enough samples, thus reducing the technical and biological variability. Despite the satisfactory model performance, spectral pre-processing such as multiplicative scatter correction, standard normal variate and others should be applied to remove or minimise variability. However, it should be handled well based on the data nature as inappropriate transformation can cause alteration to data quality.

## 4. Conclusions

The present study shows that the ATR-FTIR method is appropriate for identifying the presence of urea through its functional group. The models created through dataset refinement have demonstrated the ability to accurately classify over 90% of the test samples into their respective classes using PCA and DA models. The MLR analysis resulted in coefficients of determination of 0.996 and 0.940 for calibration and cross-validation, respectively, by utilising the significant variable from PCA and DA models. This approach yields comparable results with the previous study, indicating the relevance of this investigation. Moreover, based on the regression model analysis, it has been determined that the amide I and II regions at 1626.63, 1601.98, and 1585.5534 cm^−1^ are the most significant variables for detecting urea in milk. Thus, it can become a reference for future applications for identifying urea by looking at the substantial wavenumber selected in this study instead of the whole spectra, which can help save time and cost. The present study demonstrated that integrating ATR-FTIR with DA and MLR techniques can effectively and expeditiously identify and measure urea’s presence in milk, with a sensitivity as low as 1 *w*/*v*%, rendering it suitable for commercial applications and as a routine analysis. For recommendation, multicollinearity can be addressed using appropriate tools, effectively mitigating its impact on the model’s performance. Furthermore, the milk sample can be subjected to freeze-drying to improve the clarity of the milk spectra when addressing other adulterants that may be present in the broad water region.

## Figures and Tables

**Table 1:** List of samples including training and testing dataset.

Sample Concentration	Number of Samples	Training Dataset	Testing Dataset
Control (unadulterated milk)	25	20	5
Milk with urea (0.5–5 (*w*/*v* %))	175	140	35

**Table 2:** Classification matrix of training, validation, and testing datasets by discriminant analysis.

Dataset	Sensitivity and Specificity %	Number of UHT Milk Samples and *p*-Values of Fisher Distance		Total UHT Milk Sample
		Pure Milk	Milk + Urea	
Training dataset	Full spectra			
Pure Milk	100	11 (1)	0 (<0.0001)	11
Milk + Urea	100	0 (<0.0001)	79 (1)	79
	1675–1560 cm^−1^			
Pure Milk	55.56	5 (1)	4 (<0.0001)	9
Milk + Urea	100	0 (<0.0001)	81 (1)	81
	1585–1454 cm^−1^			
Pure milk	78.57	11 (1)	3 (<0.0001)	14
Milk + Urea	100	0 (<0.0001)	76 (1)	76
Validation dataset	Full spectra			
Pure milk	100	11 (1)	0 (<0.0001)	11
Milk + Urea	98.73	1 (<0.0001)	78 (1)	79
	1675–1560 cm^−1^			
Pure milk	44.44	4 (<0.0001)	5 (1)	9
Milk +Urea	97.53	2 (<0.0001)	79 (1)	81
	1585–1454 cm^−1^			
Pure milk	78.57	11(1)	3 (<0.0001)	14
Milk + Urea	98.68	1 (<0.0001)	75 (1)	76
Testing dataset	Full Spectra			
Pure milk	100	5 (1)	0 (<0.0001)	5
Milk + Urea	100	0 (<0.0001)	35 (1)	35
	1675–1560 cm^−1^			
Pure milk	40	2 (<0.0001)	3 (1)	5
Milk + Urea	91.43	3 (<0.0001)	32 (1)	35
	1585–1454 cm^−1^			
Pure milk	80	4 (1)	1 (<0.0001)	5
Milk + urea	94.29	2 (<0.0001)	33 (1)	35

Notes: *p*-value of Fisher distance < 0.05 indicated two groups were significantly different. Full spectra represent wavenumber 4000–500 cm^−1^.

**Table 3:** Effect of different spectral windows on spectral data modelling using MLR for the selected range of wavenumbers.

Wavenumber Range (cm^−1^)	Calibration			Validation			Most Significant Wavenumber
	R^2^	RMSEC	MSE	R^2^	RMSE	MSE	
Full spectra	0.996	0.171	0.029	0.940	0.941	0.886	1626.63 cm^−1^
1675–1560	0.991	0.170	0.029	0.993	0.168	0.028	1601.98 cm^−1^
1584–1453	0.988	0.203	0.041	0.981	0.303	0.092	1585.5534 cm^−1^

R^2^: Determination of coefficient, MSE: mean square error; RMSE: root mean square error.

**Table 4:** Determination for the testing dataset of known urea adulteration percentage in UHT milk.

Wavenumber cm^−1^	Actual Urea Adulteration (%)	Determined Urea Concentration (%) ± SD	95% Lower and Upper Bounds of Determined Urea Adulteration	*t*-Test Value
Full spectra	0	0.027 ± 0.287	−0.324–0.377	0.857
	0.5	0.272 ± 0.063	−0.066–0.610	<0.0001
	1	1.038 ± 0.103	0.734–1.342	0.483
	1.8	1.686 ± 0.189	1.365–2.006	0.261
	2.6	2.499 ± 0.208	2.168–2.931	0.358
	3.4	3.299 ± 0.287	2.994–3.604	0.503
	4.2	4.199 ± 0.571	3.694–4.704	0.997
	5	5.691 ± 0.112	5.255–6.127	<0.0001
1675–1560	0	−0.249 ± 0.100	−0.269–(−0.146)	<0.0001
	0.5	0.263 ± 0.024	0.151–0.375	<0.0001
	1	1.065 ± 0.051	0.972–1.159	0.002
	1.8	1.842 ± 0.060	1.735–1.949	0.158
	2.6	2.498 ± 0.190	2.387–2.610	0.268
	3.4	3.201 ± 0.306	3.061–3.341	0.183
	4.2	4.118 ± 0.434	3.928–4.309	0.686
	5	5.865 ± 0.247	5.722–6.007	<0.0001
1585–1485	0	−0.401 ± 0.104	−0.573–(−0.229)	<0.0001
	0.5	0.071 ± 0.214	−0.145–0.286	0.002
	1	1.075 ± 0.116	0.918–1.231	0.190
	1.8	1.815 ± 0.186	1.612–2.018	0.860
	2.6	2.701 ± 0.191	2.541–2.861	0.271
	3.4	3.225 ± 0.569	3.013–3.436	0.510
	4.2	4.306 ± 0.394	3.967–4.645	0.565
	5	5.962 ± 0.513	5.747–6.176	0.003

Note: Urea concentration expressed as means ± standard deviation of twenty sample replications.

## Data Availability

The data presented in this study are available on request from the corresponding author.
